# Parental attitudes to genetic testing differ by ethnicity and immigration in childhood nephrotic syndrome: a cross-sectional study

**DOI:** 10.1186/s40697-016-0104-y

**Published:** 2016-03-17

**Authors:** Karlota Borges, Jovanka Vasilevska-Ristovska, Neesha Hussain-Shamsy, Viral Patel, Tonny Banh, Diane Hebert, Rachel J. Pearl, Seetha Radhakrishnan, Tino D. Piscione, Christoph P. B. Licht, Valerie Langlois, Leo Levin, Lisa Strug, Rulan S. Parekh

**Affiliations:** Child Health Evaluative Sciences, Research Institute, The Hospital for Sick Children, Toronto, Canada; Division of Nephrology, The Hospital for Sick Children, 555 University Avenue, Toronto, Ontario M5G 1X8 Canada; University of Toronto, Toronto, Canada; Dalla Lana School of Public Health, Toronto, Canada

**Keywords:** Genetic testing, Decision-making, Nephrotic syndrome, Caregiver, Steroid resistant nephrotic syndrome, Pediatric

## Abstract

**Background:**

Studies in the USA report differences in opinion among parents of different ethnic groups toward genetic testing for their child; however, there are no studies that address this issue in the diverse ethnic and immigrant population in Canada.

**Objective:**

This study aims to determine whether ethnicity and immigration status influences parental interest in clinical genetic testing for a potentially progressive kidney disease.

**Design:**

This is a cross-sectional study.

**Setting:**

Participants were recruited from the Greater Toronto Area, Canada.

**Participants:**

The study included 320 parents of children ages 1–18 years with nephrotic syndrome enrolled in the Insight into Nephrotic Syndrome: Investigating Genes, Health and Therapeutics (INSIGHT) observational cohort study.

**Measurements:**

Demographic, ethnicity, immigration, and child specific factors as well as interest in genetic testing were collected through self-reported questionnaires administered at baseline study visit.

**Methods:**

Logistic regression models were used to examine association of ethnicity and immigration status with interest in genetic testing.

**Results:**

The majority of parents (85 %) were interested in genetic testing for their child. South Asian and East/Southeast Asian parents had 74 and 76 % lower odds of agreeing to genetic testing when compared to Europeans (odds ratio (OR) 0.26, 95 % confidence interval (CI) 0.10–0.68; OR 0.24, 95 % CI 0.07–0.79, respectively) after controlling for age and sex of child, age and education level of parent, initial steroid resistance, and duration of time in Canada. Immigrants to Canada also had significantly lower odds (OR 0.29, 95 % CI 0.12–0.72) of agreeing to genetic testing after similar adjustment. Higher education level was not associated with greater interest in genetic testing (OR 1.24, 95 % CI 0.64–2.42).

**Limitations:**

Participants have already agreed to aggregate genetic testing for research purposes as part of enrolment in INSIGHT study.

**Conclusion:**

While majority of parents were interested in genetic testing for their child, immigrants, particularly South Asians and East/Southeast Asians, were more likely to decline genetic testing. Genetic counseling needs to be tailored to address specific concerns in these parental groups to maximize informed decision-making in the clinical setting.

**Trial registration:**

ClinicalTrials.gov, NCT01605266

## Abrégé

**Mise en contexte:**

Des études aux États-Unis font état de différences d’opinions parmi les parents provenant de différentes origines ethniques quant à la possibilité de procéder à des tests de dépistage génétique sur leurs enfants. Toutefois il n’existe aucune étude qui traite de cette question au sein des différents groupes ethniques au Canada.

**Objectifs de l’étude:**

L’étude avait pour but de vérifier si l’origine ethnique ou le statut d’immigrant des parents influençait leur façon d’aborder la question du dépistage génétique pour la détection d’une néphropathie potentiellement évolutive chez leur enfant.

**Cadre et type d’étude:**

Cette étude transversale s’est tenue dans la grande région de Toronto au Canada.

**Participants:**

Il s’agit de 320 parents d’enfants âgés de 1 à 18 ans atteints d’un syndrome néphrotique qui participaient à l’étude de cohorte observationnelle INSIGHT (*Insight into Nephrotic Syndrome: Investigating Genes Health and Therapeutics*).

**Mesures:**

Les données soit la répartition démographique l’origine ethnique ou le statut d’immigrant des parents, les éléments propres à l’enfant ainsi que le niveau d’intérêt des parents à l’égard des tests de dépistage génétique, ont été colligées à partir d’un questionnaire remis aux parents lors de la première visite.

**Méthodologie:**

Des modèles de régression logistique ont été utilisés pour établir un parallèle entre l’origine ethnique ou le statut d’immigrant d’un parent et son intérêt à soumettre son enfant à un dépistage génétique.

**Résultats:**

La majorité des participants à l’étude (85 %) démontrait un intérêt envers la possibilité de soumettre leur enfant à un test de dépistage génétique. Toutefois dans le cas précis des gens originaires de l’Asie du Sud et de ceux provenant de l’Extrême-Orient ou de l’Asie du Sud-Est, les probabilités de consentir à un tel test étaient respectivement de 74 % et de 76 % plus faibles que pour les gens d’origine européenne. (Risque relatif [RR] : 0,26 à 95 % d’intervalle de confiance [IC] : 0.10-0.68; RR : 0,24 à 95 % IC : 0.07-0.79 respectivement). Ces résultats ont été obtenus après l’exclusion d’indicateurs relatifs à l’âge et au sexe de l’enfant, au sexe et au niveau d’éducation des parents, à la résistance de l’enfant au traitement initial par les stéroïdes et à la durée du séjour au Canada. Cette observation s’est également confirmée chez les immigrants reçus, pour qui la probabilité de consentir à un tel test pour leur enfant s’est aussi avérée significativement moins élevée après l’application des mêmes correctifs (RR : 0,29, à 95 % IC : 0.12-0.72). Aucune corrélation n’a pu être établie entre le niveau d’éducation élevé des parents et un intérêt accru à soumettre leur enfant à un test de dépistage génétique (RR : 1,24 à 95 % IC : 0.64-2.42).

**Limites de l’étude:**

Les résultats sont limités du fait que les participants avaient consenti à soumettre leur enfant à un test de dépistage génétique fà des fins de recherche dans le cadre de leur inclusion à l’étude INSIGHT.

**Conclusions:**

Alors que la majorité des parents ayant participé à l’étude voyait d’un bon œil la possibilité de soumettre leur enfant à un test de dépistage génétique les immigrants reçus ainsi que les participants originaires de l’Asie du Sud, de l’Extrême-Orient et de l’Asie du Sud-Est se sont avérés plus susceptibles de décliner l’offre. Par conséquent, le processus de consultation en génétique doit être adapté pour mieux répondre aux inquiétudes et aux préoccupations de ces groupes de parents; ceci afin de tirer le meilleur parti d’une prise de décision éclairée dans un contexte clinique.

## What was known before

Studies in the USA have reported differences in parental attitudes toward genetic testing among different ethnic groups, but this remains unexplored in Canada.

## What this study adds 

Immigrants, particularly South Asians and East/Southeast Asians, have significantly lower rates of agreeing to genetic testing, suggesting specific concerns about genetic testing that need to be addressed when counseling families.

## Background

Genetic testing has quickly become a clinically applied tool in medicine. It can aid in diagnosis, prevention, and treatment of disease, as well as uncover disease risk in family members. It is especially useful in the early diagnosis of children with progressive diseases so that intervention can begin as soon as possible or medications may be withdrawn that will not alter the clinical course. The use of genetic testing, however, is not universally embraced among parents.

Studies in the USA report both ethnic and cultural differences in genetic testing. Reports demonstrate that African Americans were less likely to see the potential benefits of predictive testing for disease risk and were more concerned about potential discrimination resulting from genetic testing compared to European Americans [1, 2]. Similarly, in a study on childhood deafness, Hispanic and Asian American parents were more likely to foresee harmful outcomes of genetic testing on their children compared to European parents [3]. These studies, however, have limited generalizability to Canada, as the ethnic populations as well as recent immigration patterns differ significantly between the two countries. The two main ethnic minority groups in Canada are South Asians (from India, Pakistan, and Sri Lanka) and East/Southeast Asians (from China, Japan, Korea, Vietnam, and the Philippines) [4], which are relatively under-represented in US studies. As well, there has been a shift toward more highly educated and skilled immigrants coming to Canada in the recent years, and a relatively low proportion of refugees, which also contributes to the differing patterns of immigrants and ethnicities [5].

In Canada, people of visible minorities are less likely to use hospital or cancer screening services than non-minorities despite the public health-care systems [5]. Studies on specific ethnic groups in Canada have found that both South Asian and Chinese immigrants have poorer health status due to negative personal attitudes toward using health services [6, 7]. These negative attitudes may extend to the use of genetic testing in health-care settings, especially in parental decisions for their child. There are currently no Canadian studies that address the differences in parental attitudes toward genetic testing among cultural groups.

Nephrotic syndrome is the most commonly diagnosed kidney disease in children with an incidence that is much higher among children of South Asian ancestry in Ontario [8]. The disease is characterized by heavy proteinuria, hypoalbuminemia, and hyperlipidemia and is treated with steroids for initial therapy and relapses [9]. Most respond to steroids and have a good prognosis with preserved renal function over the long term [10]. About 10–20 % of cases, however, are steroid resistant and have a much poorer prognosis, often progressing to end-stage renal disease and requiring dialysis or a kidney transplant within 10 years of diagnosis [11–13]. Genetic testing is often suggested in those with steroid resistant disease or biopsy-proven focal segmental glomerulosclerosis, and some advocate testing early in the disease course as steroid medications may be discontinued in the presence of genetically related disease [11, 14, 15]. This remains controversial as some children with underlying genetic disorders have completely responded to steroids [16].

In an established cohort study, we asked parents about their interest in testing their child with nephrotic syndrome for a single gene associated with disease response and/or progression of kidney disease. Our objective was to determine whether ethnicity and immigration influences parental decisions toward clinical genetic testing. As genetic discovery advances, genetic testing is becoming more accessible and increasingly recommended in clinical care for nephrotic syndrome and many other diseases. Understanding the concerns and opinions of parents toward genetic testing will allow health professionals to better facilitate clinical decision-making.

## Methods

### Study population and design

The data reported are collected from questionnaires completed by parents enrolled in the Insight into Nephrotic Syndrome: Investigating Genes, Health and Therapeutics (INSIGHT) study (ClinicalTrials.gov Identifier NCT01605266, registered April 20, 2012). INSIGHT is an observational, longitudinal cohort study based in the Greater Toronto Area in Canada [17]. The study includes children diagnosed with nephrotic syndrome between the ages of 1 and 18, excluding those with congenital nephrotic syndrome, those with multiple organ involvement, and those with secondary causes of nephrotic syndrome including those with systemic lupus erythematosus or vasculitis.

A primary aim of the INSIGHT study is to understand genetic factors associated with disease susceptibility and progression. Participants consent for the INSIGHT study to collect and store samples for future studies on nephrotic syndrome and other kidney-related research. All participants are informed that samples are de-identified and studied in aggregate and as such, families will not be informed of their individual child’s genetic test results. Consent forms in languages other than English are also available if needed. As part of the study, parents complete questionnaires in English with trained research staff and with certified translators (or the child when appropriate) if the participant is not fluent in English. Informed consent is obtained from both parents and children, and the study is approved by the Hospital for Sick Children’s Research Ethics Board.

This study is a cross-sectional analysis of data from 320 parents of the 320 children enrolled in INSIGHT that completed the baseline study visit questionnaire.

### Measurements

The questionnaires included information on demographics, self-reported ethnicity, immigration history, highest level of education achieved, family income, language spoken at home, and family history of chronic diseases. Parental interest in genetic testing was assessed by the following question in the survey: *There is a possibility that a specific gene may be associated with frequent relapses of nephrotic syndrome, steroid responsiveness and progression of kidney disease; if your child could be tested for this gene, would you be interested?* Using a standardized script, the research coordinators told parents that this was a hypothetical question to assess interest in genetic testing for a single-specific nephrotic syndrome gene and that results of the test could theoretically alter the treatment that their child would receive (steroids or another agent) or be able to identify whether their child was at increased risk of disease progression. Choices included “yes,” “no,” and “uncertain,” and the last two were grouped together for analyses. If parents were interested in genetic testing, they were asked to indicate their reasons for interest in genetic testing and instructed to select one or more options from a list of seven reasons, and parents that were uncertain or not interested were re-contacted and asked to provide their reasoning. In INSIGHT, nine coordinators of different ethnicities implemented the questionnaires.

### Statistical analyses

Descriptive statistics were examined for normality and compared with *χ*^2^, *t* tests, and Fisher’s exact tests by status of interest in genetic testing. Using logistic regression, we determined whether ethnicity or immigration were associated with interest in genetic testing adjusting for demographic, education, or child specific factors. Parent ethnicity and immigration status were then analyzed using multivariable analyses in two parsimonious models adjusting for age and education level of parent, age and sex of child, initial steroid resistance, and duration of time in Canada. Data were checked to ensure covariates had linear association with log odds of genetic testing, and all models were examined for multicollinearity, specification, and goodness of fit to ensure assumptions of the models were met. Statistical analyses were conducted using STATA/SE-14, and a *p* value <0.05 was considered significant.

## Results

We screened over 550 children and 70 % of eligible children and families consented to participate in the INSIGHT study (5 % are still undecided and 25 % have refused). Those that refused to participate were mainly of South Asian (45 %), East/Southeast Asian (15 %), or European descent (15 %). There were no differences in age or sex of those who consented compared to that did not consent (data not shown). Table 1 shows the baseline characteristics of 320 parents of INSIGHT participants by interest in genetic testing. Parents were mainly of European and South Asian origin and had a high level of education overall (73 % with post-secondary education or higher). Children were a median age of 9.4 years at the time of questionnaire completion and were predominantly male.

**Table 1:** Baseline characteristics of 320 INSIGHT participants by parental interest in genetic testing

		Parental interest in genetic testing		*p* value
	Total (*n* = 320)	Yes (*n* = 271)	No/uncertain (*n* = 49)	
	*n* (%) or mean ± SD or median (interquartile range)			
Parent characteristics				
Age (years) (*n* = 297)	41.4 ± 7.4	41.8 ± 7.4	39.0 ± 6.6	0.01
Education level				0.5
Primary/high school	86 (26.9)	71 (26.2)	15 (30.6)	
University/graduate	234 (73.1)	200 (73.8)	34 (69.4)	
Self-reported ethnicity (*n* = 319)				<0.001
European	121 (37.9)	111 (41.1)	10 (20.4)	
South Asian	112 (35.1)	84 (31.1)	28 (57.1)	
East/Southeast Asian	28 (8.8)	21 (7.8)	7 (14.3)	
Other^a^	58 (18.2)	54 (20.0)	4 (8.2)	
Immigration status (*n* = 319)				<0.001
Born in Canada	131 (41.1)	123 (45.6)	8 (16.3)	
Born outside Canada	188 (58.9)	147 (54.4)	41 (83.7)	
Duration of time in Canada (*n* = 307)				0.08
>10 years	257 (83.7)	225 (85.2)	32 (74.4)	
≤10 years	50 (16.3)	39 (14.8)	11 (25.6)	
Language spoken at home (*n* = 316)				0.009
English only	204 (64.6)	183 (68.0)	21 (44.7)	
English and other language	26 (8.2)	20 (7.4)	6 (12.8)	
Other language only	86 (27.2)	66 (24.6)	20 (40.5)	
Child characteristics				
Male children	202 (63.1)	170 (62.7)	32 (65.3)	0.7
Child age (years)	9.4 [5.6–15.0]	10.5 [6.0–15.2]	7.0 [4.9–10.3]	0.01
Family history of kidney disease	99 (30.9)	88 (32.5)	11 (22.5)	0.2
Family history of CVD^b^, diabetes, or hypertension	100 (31.3)	90 (33.2)	10 (20.4)	0.08
Initial steroid resistance	20 (6.4)	16 (6.1)	4 (8.3)	0.5

*p* values are calculated using χ^2^, *t* test, or Fisher’s exact test. Age is calculated at time of questionnaire completion. Variables with incomplete information for all 320 parents have population stated in brackets

^a^Other includes Middle Eastern, West Indian/Caribbean and African, Mexican/South and Central American, Aboriginal, and Multi-ethnic

^b^Cardiovascular disease

Eighty-five percent of parents were interested in genetic testing for their child. Of the remaining parents, 32 (10 %) were unsure and 17 (5 %) not interested in genetic testing, and these parents tended to be younger and have very young children. These parents were also mainly of South Asian origin, born outside of Canada, recently immigrated, and spoke a language other than English at home.

Several factors were found to be significant predictors of parental interest in genetic testing by univariable analyses: European ethnicity, being born in Canada, older age of parent, English spoken at home, and older age of the child (Table 2). Both South Asians and East/Southeast Asians had 73 % lower odds of agreeing to genetic testing compared to Europeans. Immigrants to Canada had 77 % lower odds of agreeing to genetic testing compared to parents born in Canada, and those that spoke a language other than English at home had 63 % lower odds of agreeing to genetic testing than English speakers. French made up only 2 % of the languages spoken at home in the “other languages” category, with the majority being South Asian languages (62 %; Urdu, Tamil, Punjabi, Gujarati, Hindi, Bengali, Malayalam) and East/Southeast Asian languages (11 %; Mandarin, Cantonese, Tagalog). Older parents with older children at time of questionnaire completion had higher rates of agreement for genetic testing.

**Table 2:** Odds of higher interest in genetic testing by socio-demographic and clinical factors among 320 INSIGHT participants

	Parental interest in genetic testing					
	Unadjusted		Adjusted model 1		Adjusted model 2	
	OR	[95 % CI]	OR	[95 % CI]	OR	[95 % CI]
Parent age (per 5 years)	1.31*	[1.05–1.60]	1.30	[0.92–1.84]	1.29	[0.93–1.79]
Parent education level						
Primary/high school	ref	–	ref	–	ref	–
University/graduate	1.24	[0.64–2.42]	1.19	[0.54–2.60]	1.02	[0.47–2.20]
Parent self-reported ethnicity						
European	ref	–	ref	–		
South Asian	0.27*	[0.12–0.59]	0.32*	[0.13–0.82]		
East/Southeast Asian	0.27*	[0.09–0.79]	0.22*	[0.07–0.72]		
Other^a^	1.22	[0.36–4.06]	1.81	[0.35–9.22]		
Parent immigration status						
Born in Canada	ref	–			ref	–
Born outside of Canada	0.23**	[0.11–0.52]			0.34*	[0.14–0.83]
Language spoken at home						
English	ref	–				
English and other language	0.38	[0.14–1.06]				
Other language only	0.38*	[0.19–0.74]				
Duration of time in Canada						
>10 years	ref	–				
≤10 years	0.58	[0.27–1.23]				
Female child	1.12	[0.59–2.11]				
Child age (per year)	1.07*	[1.01–1.14]	1.01	[0.92–1.11]	1.02	[0.93–1.12]
Family history of kidney disease	1.66	[0.81–3.40]				
Family history of CVD^b^, diabetes, or hypertension	1.94	[0.93–4.06]				
Initial steroid resistance	0.71	[0.23–2.23]				

Age is calculated at time of questionnaire completion. Model 1—parent ethnicity adjusted for parent age and education level, child age and sex, initial steroid resistance, and duration of time in Canada. Model 2—parent immigration status adjusted for parent age and education level, child age and sex, initial steroid resistance, and duration of time in Canada

*p ≤ 0.05; **p < 0.001

^a^Other includes Middle Eastern, West Indian/Caribbean and African, Mexican/South and Central American, Aboriginal, and Multi-ethnic

^b^Cardiovascular disease

Parental ethnicity and immigration status were both found to be the most significant predictors of interest in genetic testing and were highly correlated in this study population (97 % of South Asian parents and 96 % of East/Southeast Asians were also immigrants) so they were analyzed in two separate models (Table 2). After adjusting for age and education level of parent, age and sex of child, initial steroid resistance, and duration of time in Canada, South Asians and East/Southeast Asians remained at significantly lower odds of expressing interest in genetic testing (odds ratio (OR) 0.26, 95 % confidence interval (CI) 0.10–0.68 and OR 0.24, 95 % CI 0.07–0.79, respectively) compared to Europeans (model 1). Immigrants to Canada also had lower odds of interest in genetic testing (OR 0.29, 95 % CI 0.12–0.72) after similar adjustment (model 2). Age of parent and child was no longer significant after multivariable adjustment.

Data analyses were also stratified for family income (*n* = 186) and relationship to child (mother vs. father; *n* = 208) if available. There were no differences by either income status or by parental sex (data not shown). There was also no difference in medication adherence by genetic testing interest (data not shown).

**Fig. 1:**
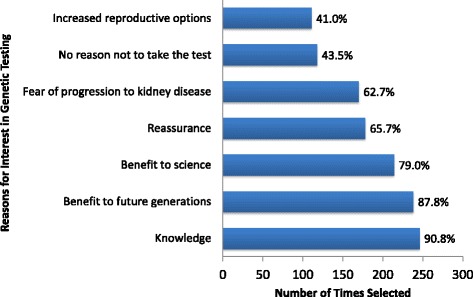
Reported reasons for interest in genetic testing among 271 interested caregivers from the INSIGHT study (multiple responses were allowed)

Parents interested in genetic testing (*n* = 271) were also asked for all the reasons influencing their interest in the future genetic testing. Knowledge, benefit to future generations, and benefit to science were the top three reasons selected (Fig. 1). Among those that were uncertain or declined genetic testing, the top reasons cited (listed in order of frequency) were a preference to wait until the child was old enough to decide for his or herself (58 %), a belief that the results would not help their child in the future (33 %), a lack of understanding at what testing involved and/or what information would be gained (21 %), beliefs that genetic testing would cost too much money and/or take too much time (21 %), desire to spare the child from further testing and blood draws (21 %), and unknown reasons in 31 % of parents that could not re-contacted.

## Discussion

Parental attitudes toward genetic testing in a chronic pediatric disease at risk of progression are significantly associated with ethnicity and immigration. Differences in parental perspectives on genetic testing of a child among ethnic groups are relatively unexplored and yet an important facet in clinical care and will continue to be an issue in Canada given our immigration patterns. Overall, 85 % of parents were interested in genetic testing. Parents that declined genetic testing were primarily immigrants to Canada and predominantly of South Asian and East/Southeast Asian descent, despite higher levels of education beyond high school. There was no concordance between medication adherence and willingness to undergo genetic testing, which suggests that these families have concerns specific to genetic testing rather than overall clinical management. It is important to understand these issues in order to help parents make informed clinical decisions for their child.

South Asian or East/Southeast Asian parents were more likely to decline genetic testing when compared to those of European ancestry, and this is independent of age and education level of the parent, age and sex of child, duration of time living in Canada, and disease severity. Reasons for these disparities could include language barriers, mistrust of the health-care system, and cultural barriers. In Canada, aging South Asian immigrants are found to have poorer physical and mental health status and yet are more uncomfortable asking for help, which is attributed in part to cultural beliefs that health problems should be kept in the family and not shared with outsiders [7]. Both South Asians and East/Southeast Asians seek outside help with health problems less frequently compared to Europeans and have less confidence in the health-care professionals’ abilities [6, 18–20]. It should be noted that the majority of South Asian and East/Southeast Asian parents in this study are first-generation immigrants, which could also add to their mistrust of Canadian health-care systems. The longer duration in Canada, however, does not necessarily increase trust. Our data demonstrate that immigrants living in Canada longer are no more likely than newer immigrants to agree to genetic testing, suggesting specific cultural issues remain even after acculturation to a Canadian lifestyle.

Among parents that declined testing, there was a strong need for more information about what genetic testing involved and concerns about what information would be obtained and whether it would help improve their child’s health. This underlines the importance of how genetic testing is framed and what information is gained in parental decision-making processes. In this study, parents were asked about genetic testing in the context of identifying a gene that may alter treatment and management, or uncover an increased risk of disease progression. As genetic testing in this clinical setting is increasingly available and even recommended for nephrotic syndrome, it is important to understand concerns or additional information required to make an informed decision.

Previous reports indicate that higher education increases interest in genetic testing. In contrast, we found that higher parent education level was not significantly associated with interest in genetic testing and did not attenuate the findings of ethnic or immigrant groups. Our study population was highly educated, and the lack of association could be due to minimal variability to demonstrate a statistical difference or could reflect that education does not alter understanding of clinical testing. We do not specify in our questionnaires the country in which parents received their education, which is a limitation. It may also be that education level and knowledge about genetic testing is not as closely linked as health-care providers suggest. Relying on education level as an indicator of health literacy may, in fact, distract from understanding parental decision-making processes.

Our study has several strengths. The population includes a large number of parents compared to prior studies of childhood chronic diseases, with detailed parental characteristics including demographics, education, ethnicity, and immigration status. It is also the only study to our knowledge that investigates parental attitudes toward genetic testing. Our study does have several limitations. The questionnaire used in the study addressed genetic testing in the context of uncovering a gene that may alter management; however, the questionnaire was not designed to address a number of evolving issues in genetic such as incidental findings from studies of the entire genome. Additionally, the questionnaire did not quantify the interest of parents by gradation but rather yes and no, which may oversimplify the issue, and parent comprehension of the survey question was not assessed. Our study population also includes those that have already agreed to research genetic studies, and even though we recruited a large proportion of our clinic population, it is possible that there would be an even greater reluctance to participate in genetic studies if all families were studied. There were a number of South Asian and East/Southeast Asian families that did not enroll in the study, and thus, our findings are conservative and may underestimate the differences observed between ethnic groups. Further studies exploring parental attitudes in more detail are warranted to understand these complex issues especially as genetic testing becomes more widely available for other childhood diseases.

## Conclusions

With the knowledge of gene-health relationships expanding rapidly, genetic testing is increasingly available and often recommended in clinical medicine. Health-care professionals must be prepared to address concerns of South Asian and East/Southeast Asian parents, particularly immigrants to Canada, about genetic testing and perhaps identify the personal barriers influencing health-care decisions. Future research should seek to understand these ethnic and immigrant-based apprehensions about genetic testing in order to provide the best parental support in clinical decision-making for their child.

## Abbreviations

CI: confidence interval
CVD: cardiovascular disease
INSIGHT: Insight into Nephrotic Syndrome: Investigating Genes, Health, and Therapeutics
OR: odds ratio
